# Exosomes and cholangiocarcinoma: mechanisms, diagnostic, and therapeutic perspectives

**DOI:** 10.3389/fimmu.2026.1779516

**Published:** 2026-03-20

**Authors:** Jing Zhang, Maoshan Chen

**Affiliations:** 1 Department of Oncology, Tongji Hospital, Tongji Medical College, Huazhong University of Science and Technology, Wuhan, China; 2 Department of Blood Transfusion, Laboratory Medicine Center, The Second Affiliated Hospital, Army Medical University, Chongqing, China; 3 Hematopoietic Acute Radiation Syndrome Medical and Pharmaceutical Basic Research Innovation Center, Ministry of Education of the People’s Republic of China, Chongqing, China

**Keywords:** cholangiocarcinoma, diagnosis, exosomes, immune evasion, immunotherapy

## Abstract

Cholangiocarcinoma (CCA) is a highly malignant solid tumor originating from the biliary tract epithelium. Due to its aggressive behavior and late clinical presentation, it poses significant diagnostic and therapeutic challenges. Therefore, finding specific biomarkers and early screening of CCA susceptible people are the current top priorities. Exosomes, a subtype of extracellular vesicles, are secreted by many cells and widely distributed in body fluids. In recent years, exosomes are known to play pivotal roles in cell communication, tumorigenesis, metastasis, and immune modulation. This review summarizes recent advances in our understanding of the relationship between exosomes and cholangiocarcinoma, with a focus on their biological functions, diagnostic implications, and therapeutic potentials.

## 1 Introduction

Cholangiocarcinoma (CCA) is a group of malignancies that originate from the bile ducts, and can be classified into intrahepatic CCA (iCCA) and extrahepatic CCA (eCCA), where eCCA can be further divided into perihilar CCA (pCCA) and distal CCA (dCCA) (1–3). The CCA incidence is relatively low in Western countries (about 8000 cases each year in the United States), but it is common and still increasing in Southeast Asia including Northeast Thailand, Korea, Japan, and China. This disease shows significant geographical variation, with higher incidences in regions where liver fluke infestation is prevalent (4, 5). More importantly, CCA is characterized by late diagnosis and poor prognosis. The five-year survival rate for advanced CCA is less than 10%, underscoring the urgent need for novel biomarkers and therapies (3, 6). Currently, the mainstay of treatment for early-stage CCA is surgery. However, most patients are diagnosed at an advanced stage, precluding surgical resection. Cisplatin plus gemcitabine combined chemotherapy with immune checkpoint inhibitors (ICIs), such as durvalumab and pembrolizumab, is the standard first-line treatment for advanced, unresectable CCA (7, 8), with the objective response rate (ORR) of up to approximately 20% (9, 10). The development of new diagnostic and therapeutic strategies is urgently needed to improve the prognosis of CCA patients.

Exosomes are small extracellular vesicles with a diameter ranging from 30 to 150 nm, which are secreted by most cells, including tumor cells (11, 12). They are able to transfer a diverse cargo of biological molecules, such as proteins, lipids, mRNAs, and non-coding RNAs from the parent cells to recipient cells, thereby influencing the biological functions of recipient cells. In the tumor microenvironment (TME), via this way of cell-to-cell communication exosomes can promote tumor cell proliferation, invasion, and metastasis by modulating the behavior of neighboring cells, including cancer- associated fibroblasts (CAFs), immune cells, and endothelial cells (13–20). For example, tumor-derived exosomes can suppress the immune response, facilitating tumor immune evasion (21, 22). Moreover, exosomes can also carry genetic and epigenetic information, which may be involved in tumorigenesis and the development of drug resistance (23–25). Exosomes are not only stable and ubiquitous in body fluids but also carry cell-specific molecules, and may become a new type of drug delivery vector, demonstrating unique advantages in the targeted therapy (26, 27). This provides the possibility for exploring exosome-related tumor markers, valuable information for early diagnosis and drug-loading treatment technologies.

There are not many review studies about exosomes in CCA, they mainly focus on the exosomal biomarkers and tumor microenvironment (2, 16, 28–31). In this review, we provide an update of the clinical applications of exosomes in CCA and extend it to their biological functions and activities in CCA tumor progression, microenvironment, immune evasion and drug resistance.

## 2 Exosomes function in CCA pathogenesis

**Figure 1:**
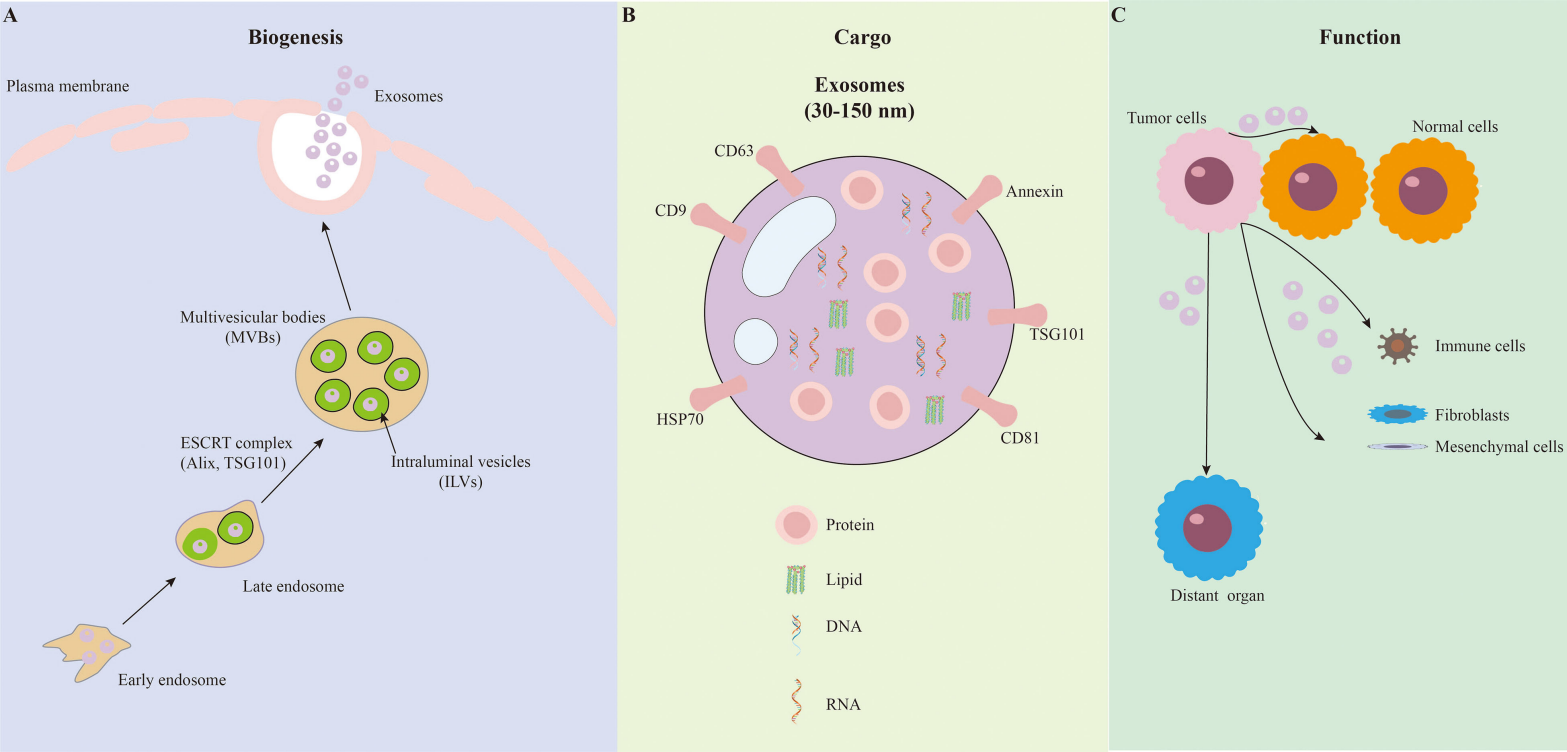
Biogenesis, cargo composition, and functional targets of exosomes in CCA. **(A)** Biogenesis of exosomes: exosome formation starts with the invagination of the plasma membrane to form early endosomes, which further mature into late endosomes. The internal membrane of late endosomes invaginates to generate intraluminal vesicles (ILVs), and late endosomes containing ILVs are defined as multivesicular bodies (MVBs). The ESCRT complex, including core components Alix and TSG101, is involved in regulating the formation of ILVs and MVBs. Finally, MVBs fuse with the plasma membrane to release ILVs into the extracellular space, which are then referred to as exosomes. **(B)** Exosomes are small extracellular vesicles with a diameter of 30–150 nm. Exosomes express specific surface markers, including CD63, CD9, CD81, Annexin, TSG101, and HSP70. Exosomes encapsulate diverse functional cargoes, including protein, lipid, DNA, and RNA, which are protected by the exosomal lipid bilayer. **(C)** Targets of CCA exosomes consist of various recipient cells, including normal cells, immune cells, fibroblasts, mesenchymal cells, and cells in distant organ, to mediate intercellular communication and participate in tumor progression and metastasis.

The biogenesis of exosomes has been reviewed very well in another study (32). Through the endosomal pathway (**Figure 1A**), exosomes can be classified via some endosome associated markers, such as CD63, CD81, SDCBP, and ESCRT complex proteins (e.g., ALIX, PDCD6IP, TSG101) (33). Also, during their biogenesis, exosomes are selectively enriched with various cellular cargoes (**Figure 1B**) which reflect the states of parental cells (31). The molecules inside exosomes are the basis of their functions, which also lead to the heterogeneity of exosomes. For example, cancer cell derived exosomes carry oncogenic molecules like neoantigens and oncoproteins (e.g., MET, MIF, EGFRvIII, CD147, PCA3, GPC1, PIGR and PD-L1) (33). When these onco-exosomes arrive at neighboring and distant cells, they can promote the progression of primary tumor or support the metastasis via modulation of various activities of recipient cells (**Figure 1C**), such as cell invasiveness, metabolic reprogramming, apoptosis, and immune suppression. In CCA, various types of cargo including oncoproteins (FZD10, claudin-3), microRNAs (miRNAs), long non-coding RNAs (lncRNAs), piwi-RNA (piRNAs) and circular RNAs (circRNAs) have been identified in the exosomes that modulate the tumor progression (34, 35). We summarized the proteins and RNA species in CCA exosomes and their functions in CCA progression (**Table 1**).

**Table 1:** Exosomal cargoes and their functions in CCA pathogenesis.

Type	Cargo	Source	Recipient cell	Function	PMID
Protein	FZD10	HUCCT-1 cells	FDZ10-mRNA silenced cells	Cancer reactivation and long-distance metastasis.	(36)
mRNA					
Protein	GAS2L3	CCA KKU-213A cells	OUMS-24/P6X cells	Promote fibroblast migration.	(37)
Protein	BMI1	BMI1-overexpressing cells (CM/LV5-BMI1)	QBC-939 and RBE cells	Promote CCA proliferation and metastasis through autocrine/paracrine mechanisms.	(38)
Protein	16 proteins including ST6GAL1	HuCCT1 and HCCC-9810 cells		Alterations in proteins involved in cancer cell adhesion and glycosylation pathways, along with specific changes in N-glycan structures.	(39)
miRNA	miR-205-5p	KKU-M213 cells		Promote cell invasion and migration.	(34)
miRNA	miR-182/183-5p	bile of CCA patients	CCA and mast cells	Promotes CCA proliferation, invasion and EMT; increasing prostaglandin E2 generation, which stimulates PTGER1 and increases CCA stemness.	(40)
miRNA	miR-141-3p	Gemcitabine resistant cell line CC-LP-1-GR	CC-LP-1	Acquire resistance to gemcitabine.	(41)
miRNA	miR-23a-3p	HUCCT-1 cells	RBE cells	Motivate cancer cells proliferation, migration and invasion.	(42)
miRNA	miR-206	iCCA-derived cancer-associated fibroblast cells	HUCCT-1 cells	Promote environment and suppress malignancy.	(43)
miRNA	miR-30e	CCA cells with miR-30e overexpression	recipient CCA cells	Suppress the proliferation, viability, invasion and migration of CCA cells via suppressing the EMT pathway.	(44)
miRNA	miR-34c	cancer-associated fibroblasts	HUCCT-1 cells	Suppress CCA progression.	(45)
LncRNA	LINC01812	CCA cells	THP-1 cells	Induce M2 macrophage polarization to promote perineural invasion.	(46)
LncRNA	LINC00152	cancer-associated fibroblasts	CCA cell lines (HIBEC, CCLP-1, HCCC-9810, HuCCT1, Huh-28, KMBC, QBC939 and RBE)	Promote cholangiocarcinoma cell proliferation, migration, and invasion.	(47)
LncRNA	TTN-AS1	CCA cell lines (Huh28, CCLP1, TFK-1, SK-ChA-1, FRH-0201, KMBC, QBC939)	normal human intrahepatic bile duct epithelial cells (HIBEC)	Bolster the metastasis tendencies of CCA cells.	(48)
LncRNA	HCG18	HCG18 overexpressed QBC939 cells	HCCC9810 cells	Accelerate tumor growth and metastasis.	(49)
CircRNA	circ-0000284	Human cholangiocarcinoma cell lines (RBE and HuCCT-1)	293T cells	Enhance the migration, invasion and proliferation of CCA cells.	(50)
CircRNA	circ-CCAC1	CCA cells	endothelial monolayer cells	Disrupt endothelial barrier integrity, induce angiogenesis, and accelerate both CCA tumorigenesis and metastasis.	(51)
CircRNA	circ_0020256	Tumor-associated M2 macrophages	CCA cells (RBE and HCCC-9810)	Promote the proliferation, migration, and invasion of CCA cells.	(52)
Lipid	sphingolipids	HUCCT-1 cells	PBMCs	Induce pro-inflammatory cytokine expression in monocytes.	(53)

Owing to the development of proteomic technologies (e.g., tandem mass spectrometry), multiple proteins (GAS2L3, FZD10, BMI1, LG3BP/MAC2BP/M2BP) in CCA exosomes have been identified to be involved in different cancer pathways (36–39, 54), such as fibroblast migration, wnt signaling activation, CCA proliferation/metastasis, and normal biliary epithelial cell malignant transformation (via β-catenin upregulation and E-cadherin downregulation). In addition, PTEN is reported to act as a critical switch for CCA exosome secretion, with its deficiency disrupting lysosome biogenesis to boost exosome release and cancer metastasis (55). This links intracellular signaling (lysosome biogenesis) to intercellular communication (exosome release), revealing a novel oncogenic mechanism of PTEN deficiency in CCA – beyond its classic function as a tumor suppressor in the PI3K/AKT pathway (56). On the other hand, exosome secretion is reduced in IDH1 (isocitrate dehydrogenase 1)-mutated CCA (57), potentially linked to the high expression of the ATP receptor P2RX7 (a regulator of exosome release/localization), representing the first research into exosomes in IDH1-mutated CCA. Actually, exosomal protein research in CCA remains limited, but proteomics advancements are driving growing attention to their functional roles.

Various RNA types including mRNA, lncRNA, miRNA and circRNA have been reported to function in CCA pathogenesis (**Table 1**). Among them, miRNAs are the most well-studied ncRNAs in CCA exosomes and their dual functional roles (oncogenic vs. tumor-suppressive) reflect the complexity of exosome-mediated intercellular communication in CCA. Oncogenic exosomal miRNAs (miR-182/183-5p, miR-205-5p, miR-141-3p) drive CCA progression through multiple complementary mechanisms, such as regulating tumor cell intrinsic properties (EMT, stemness), remodeling the tumor microenvironment (angiogenesis, mast cell activation), and mediating chemoresistance. For instance, miR-182/183-5p not only targets HPGD to enhance PGE2 and VEGF-A release—key mediators of inflammation and angiogenesis—but also promotes tumor stemness via PTGER1, creating a pro-tumor niche that supports sustained growth and metastasis (40). Similarly, the dual role of miR-141-3p in EMT induction and gemcitabine resistance highlights its clinical relevance, as EMT and chemoresistance are major barriers to CCA treatment (41, 58). Conversely, tumor-suppressive exosomal miRNAs (miR-30e, miR-206, miR-34c) counteract CCA progression, often by targeting pro-oncogenic pathways (e.g., snail-mediated EMT) or regulating stromal cell function (e.g., CAF activation) (43–45). Notably, the source of these exosomes (e.g., CAFs for miR-206) emphasizes the bidirectional crosstalk between CCA cells and the tumor stroma: while CCA cells secrete oncogenic exosomes to activate stromal cells, stromal cells can also secrete tumor-suppressive exosomes to limit tumor progression – a balance that may dictate CCA aggressiveness (43). The downregulation of exosomal miR-34c in CCA further supports this idea, as its loss removes a brake on CAF activation, creating a feed-forward loop that accelerates tumor growth (45). These findings align with broader cancer exosome research, where exosomal miRNAs often act as “molecular messengers” to propagate oncogenic signals or suppress anti-tumor pathways. For CCA specifically, the detection of miR-182/183-5p in bile exosomes (45) is particularly promising, as bile is a readily accessible body fluid—making these miRNAs potential non-invasive biomarkers for CCA diagnosis and prognosis.

Compared to exosomal miRNAs, research on exosomal lncRNAs and circRNAs in CCA is still emerging but rapidly expanding, driven by advances in next-generation sequencing (59). Exosomal lncRNAs (e.g., LINC01812, LINC00152, TTN-AS1 and HCG18) primarily function as oncogenic drivers, though their specific molecular mechanisms, such as acting as competing endogenous RNAs (ceRNAs), scaffold molecules, and regulators of transcription, remain largely uncharacterized (46–49). The identification of 54 deregulated lncRNAs in CCA bile exosomes provides a valuable resource for future studies (59), but the proposed association between ENST00000588480.1/ENST00000517758.1 and the p53 pathway requires rigorous validation – highlighting a key limitation of current lncRNA research in CCA: descriptive studies outpace mechanistic investigations. Exosomal circRNAs, by contrast, have a well-defined core mechanism – miRNA sponging (60). Circ-CCAC1, circ-0000284, and circ_0020256 all sponge specific miRNAs to upregulate pro-oncogenic target genes, thereby promoting angiogenesis, cell proliferation, and invasion (50–52). Notably, circ_0020256 is derived from tumor-associated M2 macrophages (TAMs) (52), underscoring the role of immune cells in shaping CCA progression via exosomal circRNA transfer – an understudied area that merits further exploration. A key advantage of exosomal lncRNAs and circRNAs over miRNAs is their tissue- and cancer-type specificity, which could reduce off-target effects in therapeutic applications. Additionally, their stability in body fluids (e.g., bile, serum) makes them attractive biomarker candidates, complementing exosomal miRNAs in CCA liquid biopsies.

The decreased levels of all detected sphingolipids, including Cer, DHCer, SM, hexosylceramides (HexCer), lactosylceramides (LacCer) and gangliosides (GM3), in iCCA-derived exosomes compared to normal cholangiocyte exosomes highlight a critical metabolic alteration associated with iCCA pathogenesis (53). Treatment of peripheral blood mononuclear cells (PBMCs) with exosomes derived from the iCCA cell line HUCCT-1 significantly increases the proportion of CD14+ cells expressing the pro-inflammatory cytokines MIP-1α, IL-8, and IL-1α, indicating that iCCA-derived exosomes can induce pro-inflammatory cytokine expression in monocytes.

## 3 Exosomes in CCA TME, immune evasion and drug resistance

**Figure 2:**
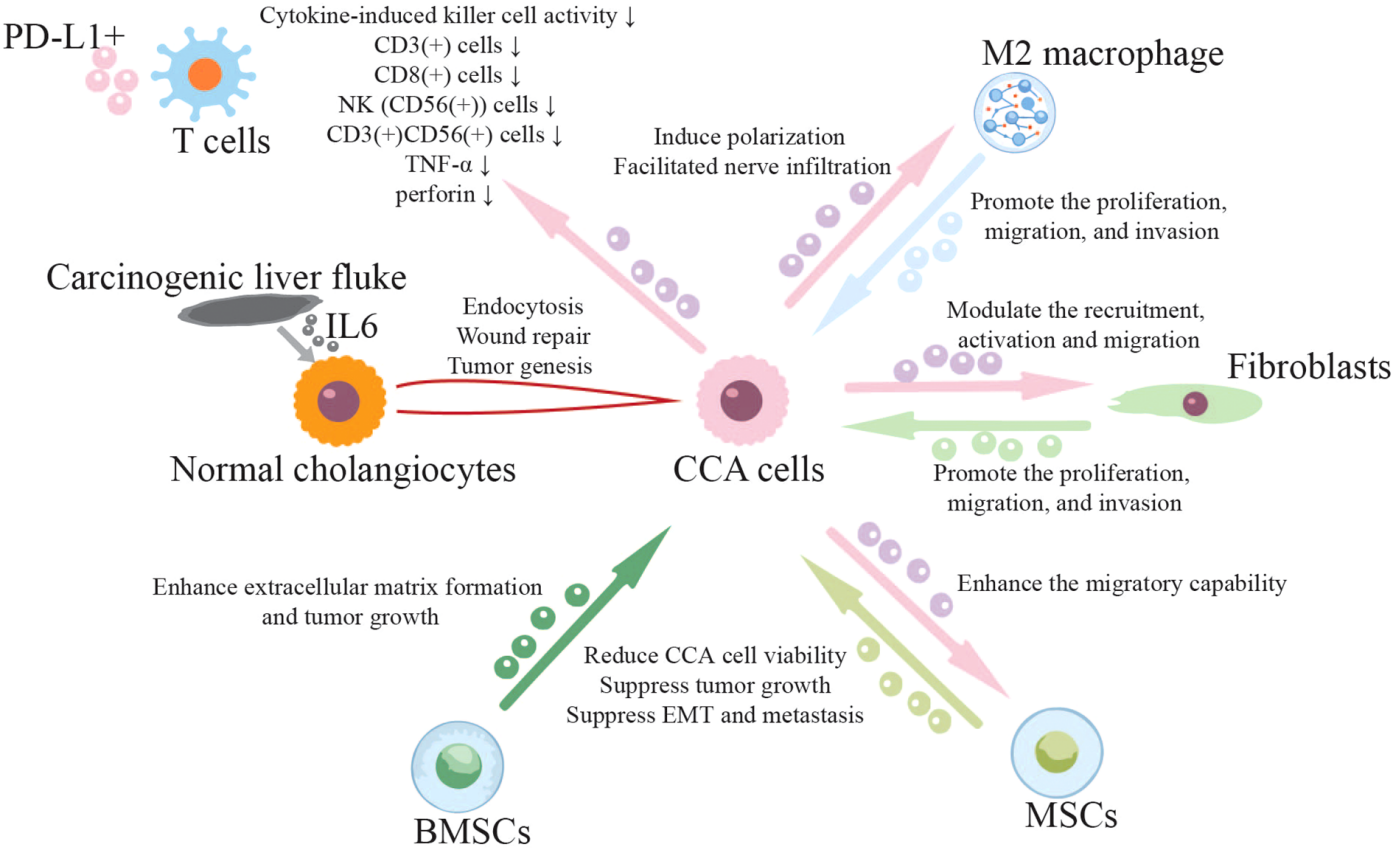
Exosomes in CCA tumor microenvironment, immune evasion and drug resistance. Exosomes play an important role in the interactions between CCA cells and other types of cells in the TAM, such as immune cells (NK, T, macrophage), fibroblasts, bone marrow stem cells (BMSCs) and mesenchymal stem cells (MSCs).

The crosstalk between CCA cells and TME (e.g., innate and adaptive cells, stromal cells, extracellular components) is a dynamic, reciprocal process mediated by both direct cellular interactions and secreted factors, including extracellular vesicles (**Figure 2**). The CCA TME is highly desmoplastic, with exosomes contributing to immunosuppression and stromal remodeling. Besides the modulators mentioned above, exosomes also transfer cytokines, chemokines and growth factors to modulate the TME of CCA. IL6, a multifaceted cytokine acting as an important mediator of immune regulation and inflammation, is transferred by carcinogenic liver fluke secreted exosomes into cholangiocytes and induces changes in protein expression associated with endocytosis, wound repair, and cancer (61). Wang et al. reported that CCA exosomes induced M2 macrophages polarization and facilitated nerve infiltration (46). In return, TAM secreted exosomes were proved to promote the proliferation, migration, and invasion of CCA cells (52). Similarly, CCA cell derived exosomes modulate the recruitment, activation and migration of fibroblasts (37, 43, 45), and CAF derived exosomes enhance the malignancy and promote CCA proliferation, migration, and invasion (47).

Stromal-epithelia cross via exosomes is also important for CCA TME. Both bone marrow stem cells (BMSCs) and mesenchymal stem cells (MSCs) are able to differentiate to CAFs. BMSC-derived exosomes enhance extracellular matrix formation and tumor growth through IL-6/STAT3 signaling in CCA cells (62). While exposure of MSCs to CCA exosomes can enhance the migratory capability of MSCs and the expression of alpha-smooth muscle actin mRNA (62). The release of CXCL-1, CCL2 and IL-6 by MSCs, in turn, can alter tumor cell proliferation. Interestingly, both BMSC- and MSC-derived exosomes are also experimented to suppress tumor growth of CCA. Increased expression of TIMP2 in CCA cells by the taken up of BMSC-derived exosomes can reduce CCA cell viability via the inhibition of key factors related to angiogenesis (VEGFA and VEGFR2) and Wnt/β-catenin pathway (β-catenin and c-Myc) (63). Exosomes from human umbilical cord MSCs are proved to suppress CCA via negatively targeting APAF1 and to suppress EMT and metastasis of CCA through targeting CHEK1 (64, 65).

Immune checkpoint inhibitors (ICIs) are currently the standard systemic treatment for CCA. However, the response rate to ICIs is relatively disappointing in CCA owing to its desmoplastic TME and immune escape (28, 66). Exosomes may play an important role in CCA immune escape. CCA exosomes subvert anti-tumor immunity by reducing cytokine-induced killer (CIK) cell activity, decreasing the population of CD3(+), CD8(+), NK [CD56(+)], CD3(+)CD56(+) cells, TNF-α and perforin secretion (67). PD-L1 expressed on cancer exosomes suppressed T cell function, and directly binded to anti-PD-L1 antibody, contributing to immune checkpoint inhibitor resistance. Also, exosomal PD-L1 may become a target for overcoming resistance to anti-PD-1/PD-L1 antibody therapy (68).

Exosomes also contribute to chemoresistance in CCA. *In vivo* studies showed that tumor cell-derived exosomes could specifically inhibit RECK (reversion-inducing-cysteine-rich protein with kazal motifs) expression via miR-210, thereby promoting the growth, metastasis, and gemcitabine chemotherapy resistance of CCA cells (69). Exosomal HSP90 (heat shock protein 90) has been successfully confirmed to be associated with the malignancy of CCA, which could stabilize mutant KRAS and promote cancer cells survival under chemotherapy pressure (70).

## 4 Clinical applications of exosomes for CCA

### 4.1 Exosomes as diagnostic biomarkers

In the past decade, exosomes have emerged as promising candidates in cancer diagnosis. Liquid biopsy utilizing exosome profiling holds significant prospects in improving early detection and monitoring of CCA. Exosomes in liquid biopsies (blood, bile) are a rich source of biomarkers. Panels of exosomal cargo (e.g., proteins like Cripto-1 and Claudin-3; RNAs like miR-200c-3p and specific circRNAs) show high accuracy for distinguishing CCA patients from healthy controls, often outperforming traditional markers like CA19-9 (**Table 2**).

**Table 2:** Exosomal cargo as potential clinical diagnostic biomarkers for CCA.

Type	Cargo	Source	Strategy	Sample size	Performance	PMID
Protein	MUC1	Serum	UV-visible signal amplification method to detect MUC1-positive exosomes using terminal deoxynucleotidyl transferase (TdT)	CCA: 11; healthy controls: 7.	Successfully distinguish CCA patients from healthy individuals.	(71)
Protein	CRP, PIGR and VWF	Serum	ELISA	Isolated PSC: 45; concomitant PSC-CCA: 44; PSC to CCA: 25; CCAs from non-PSC aetiology: 56; HCC: 34; healthy controls: 56.	non-PSC CCAs vs. healthy individuals: AUC = 0.992; OR = 387.5.	(72)
Protein	Cripto-1	Serum	exoELISA	pCCA: 115; cholangitis patients: 47; healthy controls: 65.	Sensitivity: 79.1%; specificity: 87.5%.	(73)
Protein	Claudin-3	Bile	ELISA	CCA: 10; healthy controls: 10.	AUC 0.945; sensitivity: 87.5%; specificity: 87.5%.	(35)
Protein	AMPN, PIGR	Serum	Mass spectrometry	CCA: 43; PSC: 30; HCC: 29; healthy controls: 32.	ROC of 0.878 for CCA versus control and 0.905 for CCA stage I-II versus control.	(74)
RNA	miR-194-5p and miR-192-5p	Plasma	qRT-PCR	non-Opisthorchis viverrini infected CCA: 15; non-cholangiocarcinoma subjects (healthy control): 15; O. viverrini infected subjects: 15.	miR-194-5p and miR-192-5p were upregulated in the O. viverrini-infected group; miR-192-5p was upregulated while miR-194-5p was downregulated in CCA.	(75)
CircRNA	hsa-circ-0000367, hsa-circ-0021647, and hsa-circ-0000288	Bile and serum	qRT-PCR	The Chinese Clinical Trial Registry (ChiCTR2300069863): a pilot cohort (5 CCA-BO vs. 5 BBO), a discovery cohort (10 CCA-BO vs. 10 BBO), the training cohort (113 CCA-BO and 71 BBO), and the validation cohort (51 CCA-BO and 54 BBO).	Diagnosis: Bile-DS (AUROC = 0.947); Serum-DS (AUROC = 0.861); CA19-9 (AUROC = 0.759). Prognosis: Bile-ERS (C-index=0.783); and Serum-ERS (C-index = 0.782).	(60)
RNA	miR-141-3p, miR-200a-3p, miR-200c-3p, miR-200b-3p and ENST00000588480.1	Bile and serum	qRT-PCR	Training set: 30 CCA and 30, controls; validation set: 20 CCA and 20 control.	Bile exosomal miR-200c-3p displayed the best diagnostic value with the AUC of 0.87. The combination of serum CA19–9 into the model could increase the AUC to 0.906.	(76)
RNA	miR-96-5p, miR-151a-5p, miR-191-5p, and miR-4732-3p	Blood	qRT-PCR	CCA patients: 50 GBC patients: 26; healthy control: 90.	Receiver operating characteristic (ROC) analysis for these four miRNAs showed that the corresponding AUCs were 0.733, 0.7639, 0.5417, and 0.6544, respectively.	(77)
RNA	piR-10506469	Plasma	Small RNA sequencing	CCA patients: 40; GBC patients: 25; health controls: 50.	piR-10506469 were significantly increased in the exosomes of plasma from both CCA and GBC patients.	(78)
RNA	CMIP, NME1 and CKS1B	Serum	Illumina Gene Expression Array	CCA patients: 12, PSC patients: 6; UC patients: 8; healthy controls: 9.	AUC: 0.812	(79)
RNA	UBE2C and SERPINB1	Urine	Illumina Gene Expression Array	CCA patients: 23; PSC patients: 5, UC patients: 12; healthy controls: 5.	AUC: 1.000	(79)
RNA	miR-200c-3p	Serum	qRT-PCR	CCA: 36 healthy controls: 12.	AUC: 0.93	(58)
RNA	ENST00000588480.1 and ENST00000517758.1	Bile	qRT-PCR	CCA patients (n = 35) and biliary obstruction patients (n = 56).	AUC: 0.709, sensitivity: 82.9%; specificity: 52.9%.	(59)

Mucin1 (MUC1) is a protein involved in cytoprotective and signalling pathways and is abnormally elevated in multiple cancers. Fu and colleagues developed a UV-visible signal amplification method to detect MUC1-positive exosomes in CCA patient serum, and this method successfully distinguished CCA patients from healthy individuals (71), however, the sample size of this study was relatively small. Using the ELISA method, exosomal proteins Cripto-1 and Claudin-3 enabled the detection of CCA patients from healthy controls using serum and bile as sources (35, 73). Notably, the application of more exosomal proteins would increase the prediction accuracy. For example, expression of CRP, PIGR and VWF in serum exosomes had an accuracy of separating non-primary sclerosing cholangitis (non-PSC) CCA patients from healthy individuals (72); and the expression of two proteins (AMPN, PIGR) in serum exosomes varied in CCA versus control and CCA patients at different stages (74).

RNA molecules, such as mRNA, miRNA, lncRNA, piRNA and circRNA, are another class of exosomal markers that frequently used in the clinical diagnosis of CCA (**Table 2**). As compared to current gold standard (CA19-9), the application of exosomal RNA biomarkers significantly improved the sensitivity and specificity of CCA diagnosis (**Table 2**). A single RNA molecule was approved to have the potential of being diagnostic biomarker, for example, piR-10506469 and miR-200c-3p (58, 78). Also, there are several studies reported increased accuracy of application of the combination of multiple exosomal RNAs in CCA diagnosis (59, 60, 76, 77, 79). In addition, using the exosomal RNA biomarkers CCA patients can be distinguishable from gallbladder carcinoma (GBC) patients (77, 78); miR-192-5p was found with upregulation in the plasma exosomes of both *Opisthorchis viverrini* infected and non-*Opisthorchis viverrini* infected CCA patients, as compared to healthy controls (75).

### 4.2 Therapeutic applications with exosomes for CCA

#### 4.2.1 Exosome-mediated drug delivery

**Figure 3:**
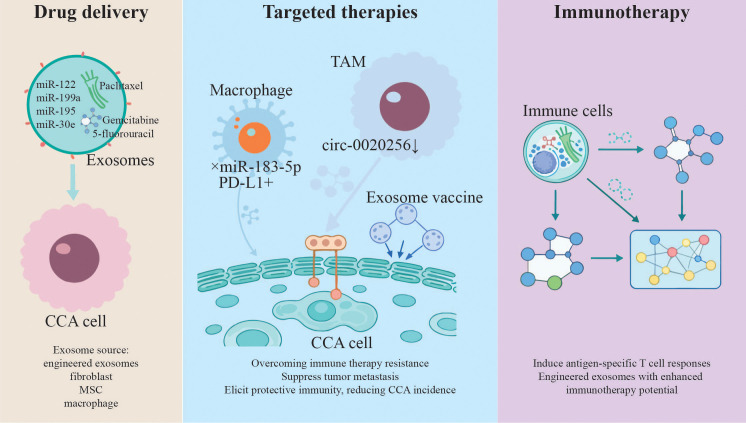
Exosomes as emerging therapeutic applications for CCA.

The biocompatibility and cargo protection of exosomes make them ideal drug carriers, transporting chemotherapeutic agents, RNA therapeutics, and proteins to target cancer cells effectively and with minimal immunogenicity (**Figure 3**). Various *in vitro* and *in vivo* studies have shown successful encapsulation and tumor-targeted delivery of anti-cancer agents via engineered exosomes in CCA models. Engineered exosomes loaded with miR-122 or miR-199a enhanced chemosensitivity in hepatocellular carcinoma by modulating mTOR and EMT pathways (80, 81). Exosome mediated miR-30e transfer could inhibit EMT by directly targeting Snail, thereby suppressing the invasion and migration of CCA cells (44). Fibroblast-derived exosomes loaded with miR-195 could be administered in a CCA rat model, concentrated within the tumor, reduced cancer size, and improved the survival rate of treated rats (82).

In preclinical models, MSC-derived exosomes delivering paclitaxel (PTX) and gemcitabine showed improved tumor penetration and reduced systemic toxicity in pancreatic cancer, suggesting translatable strategies for CCA (83). Macrophage derived exosomes loaded with the potent anticancer agent PTX represented a novel nanoformulation (exoPTX) that exhibited high anti-cancer efficacy in a mouse model of lung metastasis compared to free drug (84), suggesting translatable strategies for CCA. MSC-derived exosomes loaded with 5-fluorouracil (5-FU) enhance drug accumulation in CCA cells, overcoming chemoresistance (85). The combination of targeting ability and exosome-based drug biocompatibility provided a powerful and novel delivery platform for anti-cancer therapy.

#### 4.2.2 Exosome as targeted therapies

In addition to serving as delivery vehicles, modified exosomes can modulate the tumor microenvironment (**Figure 3**), inhibit oncogenic signaling, and stimulate anti-tumor immune responses. Blocking exosomal signaling pathways holds therapeutic promise. Inhibiting exosomal miR-183-5p in macrophages restored PD-L1 expression, potentially overcoming immune therapy resistance in intrahepatic CCA (86). Disrupting exosomal circ-0020256-mediated crosstalk between TAMs and CCA cells suppressed tumor metastasis (52). Vaccination with Opisthorchis viverrini exosomes elicits protective immunity, reducing CCA incidence in hamster models (87).

#### 4.2.3 Exosome-based immunotherapy

Harnessing exosomes as carriers of tumor antigens or immunomodulatory molecules opens new avenues in cancer immunotherapy (**Figure 3**). Clinical and preclinical investigations are exploring their use in generating specific and robust anti-CCA immune responses. Exosomes derived from immune cells regulate the immune system to generate effective anti-tumor immune responses. More importantly, exosomes can transport their cargoes to target cells, thereby affecting their phenotype and immune regulatory function. However, natural exosomes are limited in clinic due to their low drug delivery efficiency and insufficient anti-tumor ability. Technological progress enables exosomes modifications to amplify their intrinsic functions, load different therapeutic cargoes, and prioritize targeting tumor sites. The engineered exosomes have strong anti-tumor effects and great potential in cancer immunotherapy, such as restore T cell activity with expressed anti-PD-L1 antibodies in preclinical models (88). Exosomes may also become the most effective cancer vaccine. Dendritic cell (DC)-derived exosomes loaded with CCA antigens induce antigen-specific T cell responses (89).

## 5 Challenges and future directions

Despite the great potential of exosomes in CCA research, there are still several challenges and preclinical-to-clinical translation gaps that need to be addressed. One of the most promising emerging avenues in CCA exosome research lies in engineering exosomes to serve as targeted therapeutic carriers, addressing two major translation challenges: the lack of tissue specificity in unmodified exosomes and the inefficient delivery of conventional therapeutics across the blood-biliary barrier. Recent advances in exosome engineering for CCA therapy focus on three complementary strategies, each tailored to overcome unique translation hurdles. First, surface modification could enable precise targeting to CCA cells by conjugating ligands that bind to receptors overexpressed on CCA cell membranes, such as EGFR, MUC1 and FGFR4. Second, cargo encapsulation in engineered exosomes expands the scope of CCA therapeutics, addressing the drug resistance that plagues conventional monotherapies. Encapsulated cargo includes chemotherapeutic agents (e.g., gemcitabine, cisplatin), small interfering RNAs (siRNAs) targeting oncogenic drivers (e.g., KRAS, TP53), microRNAs (miRNAs) that regulate CCA progression (e.g., miR-148a, miR-125b), and immunomodulatory molecules (e.g., cytokines, checkpoint inhibitors). Third, cell source optimization is another critical engineering consideration, as the origin of exosomes influences their biocompatibility, tropism, and therapeutic potential. Modifying the parental cells of exosomes, such as MSCs, DCs, and even CCA cells, to overexpress therapeutic cargo prior to exosome isolation – has further improved preclinical efficacy. While scalable production, standardized purification, and long-term safety assessment remain challenges, engineered exosomes represent a patient-centric strategy to bridge preclinical-to-clinical gaps in CCA therapy.

A second critical emerging direction is the role of exosomes in modulating the CCA TME, a key factor in tumor immune evasion and resistance to immunotherapy – major barriers to clinical translation. The CCA TME is characterized by a highly immunosuppressive landscape, dominated by myeloid-derived suppressor cells, TAMs, regulatory T cells, and reduced infiltration of cytotoxic T lymphocytes. Exosomes have emerged as key mediators of intercellular communication within this microenvironment, transferring bioactive molecules (e.g., miRNAs, proteins, lipids) between CCA cells and immune populations, and thus shaping anti-tumor immune responses. Clarifying the molecular mechanisms of exosome-mediated crosstalk in the CCA TME will guide target discovery and immunotherapeutic development. For instance, identifying exosomal miRNAs or proteins that drive M2 TAM polarization or Treg expansion could lead to targeted inhibition strategies, while engineering exosomes to deliver checkpoint inhibitors or cytokines directly to the TME could enhance the efficacy of existing immunotherapies. This emerging direction not only addresses a critical knowledge gap but also offers a path to overcome immunotherapy resistance in CCA.

## 6 Conclusions

Exosomes have shown great potential in the field of cholangiocarcinoma research, with applications in diagnosis, prognosis assessment, treatment response monitoring, and the study of drug resistance mechanisms. The unique cargo profiles hold promise as non-invasive diagnostic markers and therapeutic vectors. Their biological contents reflect the molecular landscape of the originating tumor, providing a rich source of potential biomarkers. While exosome science holds significant diagnostic value for CCA, ongoing technical standardization and clinical validation are necessary before their routine clinical use. However, there are still many challenges that need to be overcome. Several future research directions need to be focused, including engineered exosomes for CCA therapeutics, exploring the molecular mechanisms of exosome-mediated crosstalk in the CCA TME, standardized isolation protocols, functional characterization of exosome subpopulations, clinical validation of exosome-based biomarkers and therapeutics.
